# Low-altitude economy: next frontier in spatial exploration and economic development

**DOI:** 10.1093/nsr/nwag208

**Published:** 2026-04-20

**Authors:** Heung-Yeung Shum, Shipeng Li

**Affiliations:** International Digital Economy Academy, China; International Digital Economy Academy, China

Human civilization has always advanced by conquering new frontiers—from continental discovery to space exploration. Yet the closest, most underutilized frontier lies above us: low-altitude airspace, which is typically defined below 1000 m above the ground. Low-altitude economy (LAE) consisting of all economic activities in the low-altitude airspace is projected to spawn a $9 trillion global market by 2050, according to Morgan Stanley. Following the transformative successes of high-speed rail and 5G, China is seizing this emerging opportunity in LAE to unlock a trillion-yuan domestic economic engine, redefining spatial utilization, technological innovation and national competitiveness in the process.

LAE transcends mere industrial growth; it reshapes human lives and work paradigms. As a core future transportation method, it alleviates urban congestion, cuts intercity commute time, e.g. from Shenzhen to Zhuhai in less than 20 minutes, and connects remote areas once inaccessible by traditional means, reconfiguring urban structures and boosting productivity. Its unique operational attributes extend human capabilities: drones and eVTOLs (electric vertical take-off and landing aircraft) excel in emergency rescue, forest fire suppression, infrastructure inspection and urban governance, turning once arduous or impossible tasks into efficient and safe operations. This dual value as transportation revolution and economic engine positions it as a pivotal driver of high-quality development.

The scale of low-altitude flights—dwarfing traditional aviation by several orders of magnitude—demands a paradigm shift from human-centric management to digital-driven automation, underpinned by dedicated LAE digital and physical infrastructure. Rooted in digital twin, AI and autonomous technologies, China’s intelligent low-altitude network systems, such as the Smart and Integrated Lower Airspace System (SILAS) developed by the International Digital Economy Academy in Shenzhen, enable safe, efficient oversight of heterogeneous, high-density flights. By integrating communication, navigation, surveillance and meteorological data, SILAS transforms intangible airspace into a computable resource, optimizing airspace utilization and unlocking economic potential. This fusion of cutting-edge digital technologies with aviation engineering exemplifies the seamless integration of digital and traditional economies, showcasing China’s comprehensive technological prowess. Looking ahead, SILAS and analogous systems excel in spatiotemporal computing—a core capability with immense cross-domain potential: addressing universal pain points like inefficient resource allocation and multi-agent collaboration conflicts through unified spatiotemporal modeling, dynamic optimal scheduling and rule-based autonomous decision-making, their utility extends to smart manufacturing and robot collaboration, delivering a reusable foundational spatiotemporal intelligent operating system.

At its core, LAE redefines resource value: converting idle natural airspace into a critical economic asset akin to land. Just as land use rights revolutionized societal development, airspace use rights—backed by digital technologies—emerge as a tradable, reconfigurable production factor. Unlike land, low-altitude airspace offers unparalleled flexibility: dynamically reallocated, repurposed and recycled to maximize liquidity and economic efficiency. This turns a once-dormant resource into a sustainable driver of growth.

Globally, LAE has become a battlefield for technological and rule-making leadership. China’s early strides—holding >70% of global drone patents, securing the world’s first eVTOL certifications, and launching the OpenSILAS platform—position it to set international standards for airspace management, safety protocols and industrial norms. Importantly, safety remains the cornerstone of this LAE transformation. China’s multi-layered safety system—encompassing flight safety, route security, operational oversight and social safety—ensures compliant aircraft operate safely while enabling precise detection and defeat of non-cooperative vehicles. Technologies like collision avoidance, real-time monitoring and emergency response mechanisms address inherent risks, fostering public trust and sustainable development.

For China, LAE is more than an industry; it is a testament to China’s strategic vision and technological strength. As low-altitude airspace becomes as accessible as roads, it will unlock not just economic growth, but a more efficient, equitable and connected future. China’s pursuit of this frontier is about pioneering a new era of spatial governance, cementing national rejuvenation and leading humanity’s next leap in exploring the space. Yet we must recognize that key technological bottlenecks still exist in LAE development—from high-reliability eVTOL power systems to advanced air traffic control algorithms, to name a couple. We therefore call on scientists, engineers, enterprises and policymakers to actively participate in the research and development of these core technologies, pool wisdom and resources to break through existing constraints, and jointly propel the healthy and rapid development of the LAE.

With LAE, the sky is no longer the limit.

